# An Analysis of the Use of Topical Ocular Anti-Infectives in Galicia (Spain) between 2020 and 2023

**DOI:** 10.3390/diseases12100256

**Published:** 2024-10-17

**Authors:** Severo Vázquez-Prieto, Antonio Vaamonde, Esperanza Paniagua

**Affiliations:** 1 Laboratorio de Parasitología, Departamento de Microbiología y Parasitología, Facultad de Farmacia, Universidad de Santiago de Compostela, Campus Vida, 15782 Santiago de Compostela, Spain; mesperanza.paniagua@usc.es; 2 Núcleo de Investigación en Ciencias de la Salud, Universidad Adventista de Chile, Chillán 3780000, Chile; 3 Universidad Nacional Pedro Henríquez Ureña, Santo Domingo 10203, Dominican Republic; 4 Departamento de Estadística e Investigación Operativa, Facultad de Ciencias Económicas y Empresariales, Universidad de Vigo, 36310 Vigo, Spain; vaamonde@uvigo.gal; 5 Instituto de Investigación en Análisis Químicos y Biológicos (IAQBUS), Universidad de Santiago de Compostela, 15782 Santiago de Compostela, Spain

**Keywords:** anti-infective agents, eye infections, retrospective studies, Spain, tobramycin

## Abstract

Eye infections are a global health and economic problem that affect people of both sexes at any age. Topical application of anti-infectives is widely used in the treatment of these types of infections. However, little is known about the current status and trends of the use of topical ocular anti-infectives in Spain. In the present work, we evaluated the use of this type of drug in the Spanish autonomous community of Galicia and described the variability in its consumption between Galician provinces between 2020 and 2023. In addition, the possible existence of a deviation in consumption at a seasonal level was evaluated, as well as possible changes during the study period. A descriptive, cross-sectional and retrospective study of the use of drugs belonging to the subgroups S01A (anti-infectives) and S01C (anti-inflammatory agents and anti-infectives in combination) of the Anatomic Therapeutic Chemical Classification was carried out. This work demonstrated that the most used topical ocular anti-infective in Galicia was tobramycin and that the use of these types of drugs in our region varied according to the provinces. This study also revealed that the consumption of these medications has remained stable during the period 2020–2023, with no significant seasonal differences observed.

## 1. Introduction

Eye infections can be caused by bacteria, viruses, fungi and parasites and are a global health and economic problem that affect people of both sexes at any age [1,2,3]. Topical application of anti-infectives, through the use of eye drops or ointments, is widely used in the treatment of these types of infections [4,5]. However, inappropriate use of these medications, especially overuse, can lead to the development of antimicrobial drug resistance [6,7,8] and increasing treatment failures and costs, while limiting therapeutic options [2]. Therefore, monitoring the consumption of topical ocular anti-infectives is essential to understand the patterns of their use and thus implement measures that favor their appropriate use to safeguard public health.

Little is known about the current status and trends of topical ocular anti-infectives used in Spain, and developing this understanding is essential for future stewardship programs. According to data published in our country before 2010, the consumption of topical ocular anti-infectives was almost 6 million packages per year, and a growing trend was perceived (8% since 2004) [1]. In a population registry study aiming to evaluate the use of ocular anti-infectives from 2015 to 2019 in the Spanish autonomous community of Castile and Leon, Gutiérrez-Abejón et al. [2] found an average annual dispensation of 198,000 packages of these medications and that 5.38% of the population was treated with ocular anti-infectives each year, consuming an average of 1.58 ± 1.53 packages.

The objective of this study was to evaluate the use of topical ocular anti-infectives in the Spanish autonomous community of Galicia and describe the variability in the consumption of this type of drug between its provinces between 2020 and 2023. In addition, the possible existence of a deviation in consumption at a seasonal level was evaluated, as well as possible changes during the study period.

## 2. Materials and Methods

A descriptive, cross-sectional and retrospective study was carried out on the consumption of drugs belonging to categories S01A (anti-infectives) and S01C (anti-inflammatory and anti-infectives in combination) of the Anatomic Therapeutic Chemical Classification in Galicia [9]. Galicia is the fifth autonomous community with the largest population and area in Spain; it is located in the northwest of the country and territorially structured into four provinces: Pontevedra, Ourense, Lugo and A Coruña. The information on the use of these medications was obtained through the General Subdirectorate of Pharmacy of the Galician Health Service (SERGAS) from the monthly billing database of official medical prescriptions dispensed in Galician pharmacies between January 2020 and December 2023. This database has been widely used for studies on the use of other medications in Galicia [10,11,12].

In the present study, data corresponding to the virtual medicinal product package (VMPP) have been analyzed, which is an abstract concept that provides information about the different pack sizes associated with the virtual medicinal product; that is, it is its package representation. These concepts are based on the SNOMED CTs (Systematized Nomenclature of Medicine—Clinical Terms), a structured collection of clinical terms specifically for use by healthcare professionals in the daily recording of patient care [13]. Consumption data were expressed in the number of packages dispensed, and dispensing was assumed to be equivalent to consumption. The number of dispensations/10,000 inhabitants was quantified for each year of the study period. With this, the average number of dispensations/10,000 inhabitants/year during the study period was determined. Annual population data were obtained from a publicly accessible demographic database of the Galician Institute of Statistics [14].

To process the information, a data set was created in Excel. Statistical analysis was performed using the R program (version 4.0.3). The non-parametric Kruskal–Wallis test was used to compare consumption between provinces and VMPPs. In general, a significance level of 0.05 was applied.

## 3. Results and Discussion

To the best of our knowledge, we carried out the first study that describes the use of topical ocular anti-infectives in Galician outpatients based on data obtained from community pharmacies. Drug utilization studies are essential tools to evaluate and monitor prescribing patterns and improve the quality of drug therapy. These types of studies allow data to be obtained on the patterns, quality, determinants and results of drug consumption in a studied area, with the ultimate objective of facilitating its rational use in the population [15,16,17].

**Figure 1:**
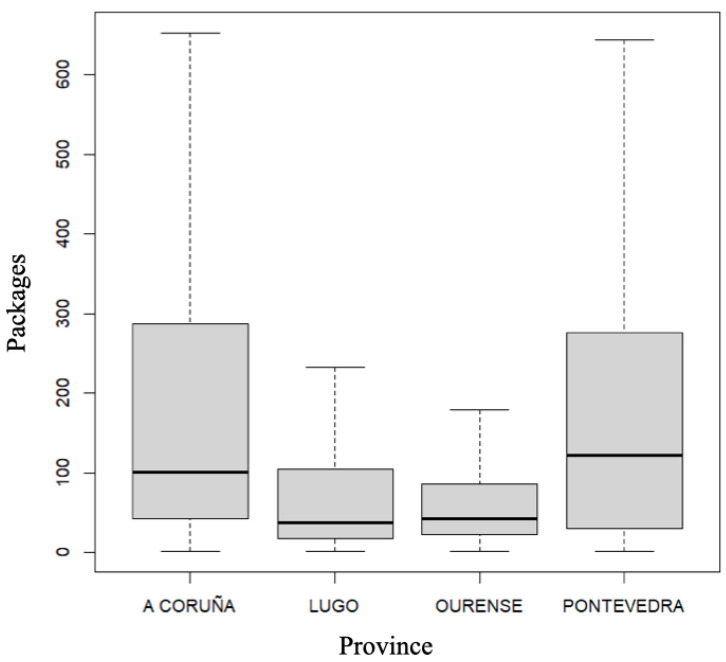
Number of packages of topical ocular anti-infectives dispensed in each province of Galicia (Spain) between 2020 and 2023.

As shown in Figure 1, the differences between provinces in terms of the number of packages dispensed were statistically significant (*p* < 0.001). Unlike the marked seasonality in antimicrobial consumption detected in other studies [18], our results did not suggest seasonality, since the differences between months were not statistically significant (*p* = 0.9867). The results obtained showed stable consumption of topical ocular anti-infectives in Galicia throughout the study period (*p* = 0.2985), including the time of the COVID-19 pandemic, which began in Spain in March 2020 (Figures S1 and S2). This result was contrary to a study conducted by Gutiérrez-Abejón et al. [2], where they noted an increase in the use of topical ocular anti-infectives in Castile and Leon, the largest region of Spain, between 2015 and 2019 (during the period covered, around one million packages of topical ocular anti-infectives were consumed, and the use of this type of medication increased by 8.23% (Z = 30.26, *p* < 0.0001, 5.17% in 2015 vs. 5.38% in 2019)). Similarly, the use of topical ocular anti-infectives has also been reported to have increased in China, where annual prescriptions for these drugs increased continuously from 126,828 prescriptions in 2013 to 163,434 prescriptions in 2019 [19]. However, other works have found a different trend in the consumption of this type of medication. For example, in a study carried out in Denmark, Norway and Sweden, with the aim of analyzing the use of topical ocular antibiotics in children aged 0 to 4 years between 2000 and 2016, the authors documented that the use of these drugs remained stable in the period 2000–2010 in all three countries and then declined [20]. Antibiotic prescribing is a complex process influenced by a combination of different factors. Thus, the stable consumption pattern observed in the present study could be related to the absence of regional variations in available resources, financial capacity and the regulation of the healthcare environment in recent years. Furthermore, other factors such as the perceptions and attitudes of medical professionals and patients, as well as the external pressure from the pharmaceutical industry, could also have remained without significant variations in the period studied [21].

**Figure 2:**
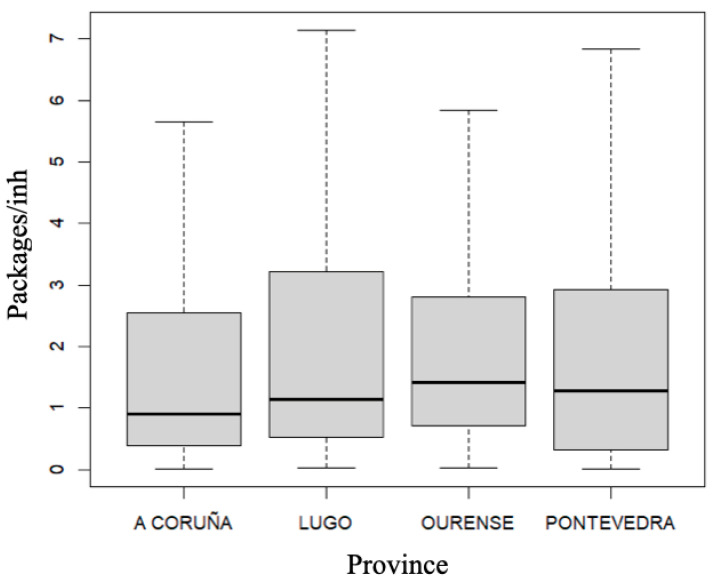
Number of packages of topical ocular anti-infectives dispensed per 10,000 inhabitants in each province of Galicia (Spain) between 2020 and 2023.

Regarding the number of packages consumed per 10,000 inhabitants (Figure 2), the differences between provinces were also significant (*p* < 0.001). The province of Ourense came in first place, followed by Pontevedra, Lugo and A Coruña. No significant differences were detected either by month (*p* = 0.9738) or by year (*p* = 0.2632), nor does a specific geographic pattern seem to be observed.

The differences found in the consumption of topical ocular anti-infectives by province may be explained by several factors. Thus, geographic variability could be related to prescribers’ preferences, adherence to clinical practice guidelines, and drug resistance and susceptibility patterns [2,19,22,23]. Similarly, the prescription of ophthalmic medications may also be associated with patient sociodemographic characteristics in each province, such as age, race, income, education level and comorbidities [24]. Furthermore, the percentage of urban population of the provinces may be another factor that contributes to differences in the use of anti-infectives [25].

**Figure 3:**
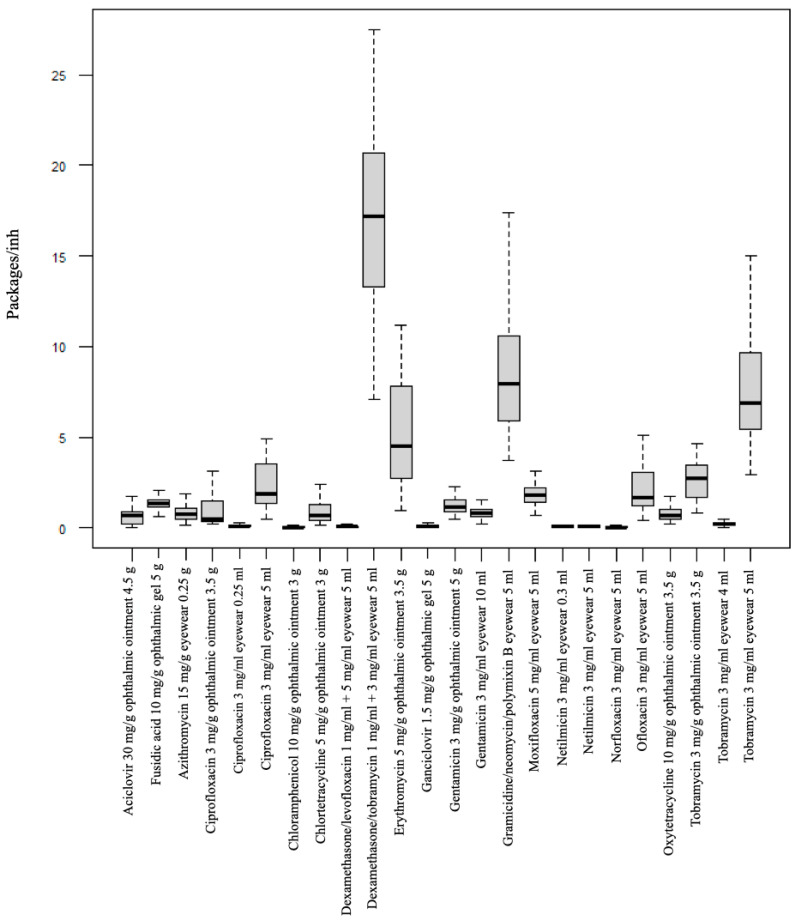
Average number of packages of topical ocular anti-infectives per 10,000 inhabitants and year dispensed in Galicia (Spain) between 2020 and 2023.

The differences between VMPPs were also significant (*p* < 0.001) (Figure 3). In the present report, the three most commonly used ophthalmic drugs were tobramycin (3 mg/mL) with dexamethasone (1 mg/mL) eye drops 5 mL (16.95), followed by gramicidin (25 IU/mL) with neomycin (1700 IU/mL) and polymyxin B (5000 IU/mL) eye drops 5 mL (8.53), and tobramycin (3 mg/mL) eye drops 5 mL (7.63). On the other hand, the three least used were levofloxacin (5 mg/mL) with dexamethasone (1 mg/mL) eye drops 5 mL (0.08), chloramphenicol (10 mg/g) ophthalmic ointment 3 g (0.07) and norfloxacin (3 mg/mL) eye drops 5 mL (0.05) (Table S1). The highest sales of tobramycin with dexamethasone corresponded to the province of Pontevedra in the months of December and November 2022 and March 2023, with a total of 2594, 2439 and 2431 packages dispensed, respectively.

Although no information was available regarding the indication of the drugs dispensed, the high levels of tobramycin in combination with dexamethasone prescribed in Galicia may be due in part to the fact that this association is used to prevent and treat inflammation and to prevent possible eye infection after a cataract surgery. It is also indicated in inflammatory processes of the eye in which there is or may be a risk of infection. On the other hand, tobramycin alone is used to treat bacterial infections of the surface and other parts of the eye, such as conjunctivitis [26]. Tobramycin inhibits protein synthesis by binding to the 16S (in the 30S subunit) and 23S (in the 50S subunit) rRNA molecules of the bacterial ribosome. It shows a bactericidal effect against a broad spectrum of Gram-positive and Gram-negative aerobic bacteria such as *Staphylococcus* spp., *Haemophilus influenzae* and *Pseudomonas aeruginosa* [27].

Gramicidin with neomycin and polymyxin B is indicated for the treatment of bacterial infections such as purulent bacterial conjunctivitis, keratitis, corneal ulcers and chronic dacryocystitis. It is also indicated in pre- and postoperative ophthalmological prophylaxis, including surgical processes and the extraction of foreign bodies from the eye [26]. This association was consumed by 0.34% of the Castilian–Leonese population between 2015 and 2019 [2]. It is noteworthy that despite the problems related to the emergence of microbial resistance [28], erythromycin and fusidic acid are the fifth and tenth most used anti-infectives, respectively.

Regarding the less used anti-infectives, it is worth mentioning that several studies have reported emerging resistance to fluoroquinolones among ocular bacterial isolates, particularly among Gram-positive organisms [29]. Consequently, a growing number of clinical guidelines advise against the use of fluoroquinolones and combinations except in the most severe infections or after treatment failure to preserve their efficacy and minimize the impact of bacterial resistance for future generations [27]. As for chloramphenicol, it inhibits bacterial protein synthesis and has a broad spectrum of action against Gram-positive and Gram-negative bacteria, including most anaerobic species. However, due to resistance and safety concerns, it is no longer used or is less frequently recommended as a first-line drug in developed countries [27].

The fact that tobramycin was the most used ophthalmological medication is in line with what was found by Gutiérrez-Abejón et al. [2] in Castile and Leon (Spain). These authors revealed that tobramycin alone or in combination with dexamethasone was consumed by more than 4% of the population and represented almost 68% of the total use of topical ocular anti-infectives, with an average of more than 135,000 packages per year. Likewise, in a nationwide study carried out between 2004 and 2008, tobramycin was the most used, representing 49.3% of the total in 2008, followed by the association of gramicidin with neomycin and polymyxin B [1]. The association between tobramycin and dexamethasone in 30% ophthalmic suspension was the most used drug in Spain in the period 2005–2007 [18]. Tobramycin was also the most commonly used agent in Belgium [30]. This increased use of tobramycin is consistent with available evidence showing that bacterial conjunctivitis is the most common ocular disorder and that this anti-infective is the most commonly used first-line medication for its treatment [31]. This contrasts with other reports, in which the use of other types of antibiotics predominated. For example, the use of polymyxin B with trimethoprim predominated in the USA [32], fusidic acid in Denmark, Sweden and the Netherlands [20,33], levofloxacin in China [19], chloramphenicol in Norway and Australia [20,34] and moxifloxacin in India [35,36]. The observed variations in antibiotic use can be attributed to various factors such as national prescribing guidelines, the characteristics of the health systems, the marketing strategies of the pharmaceutical industry, epidemiological heterogeneity in terms of the etiology of microorganisms and resistance patterns, care policies and subsidies, and the accessibility and availability of medications [2,19,22,23].

On the other hand, the results of this study show that the use of antibiotics is higher than that of antivirals, probably because infections of bacterial etiology are more common than viral ones. Thus, according to a study on acute conjunctivitis infections in children, only 12.6% of them were of viral origin, while around 80% were bacterial [37]. Acyclovir was the most widely used topical ocular antiviral drug in this study. It is the treatment of choice for keratitis caused by the herpes simplex virus and is less expensive than ganciclovir, which is the most widely used topical anti-infective in ocular viral infections in China [19].

To our knowledge, this work is the first to investigate the use of topical ocular anti-infectives in Galicia based on data from prescriptions dispensed in community pharmacies. The data obtained can be useful for comparative evaluation purposes at a temporal level within our region or geographically between Spanish regions. This brief report provides an overview of the use of topical ocular anti-infectives in Galicia as a first step in determining where stewardship efforts should be focused.

Some limitations were noted when interpreting our findings. First, because some patients stop their treatment before completing it, not all marketed products are used [1]. Second, there is no information on the use of topical ocular anti-infectives in hospitals and other settings (such as private offices) in the database examined. Third, the percentage of these types of ophthalmic agents that can be accessed without a prescription in the studied populations has not been investigated. Finally, since clinical data such as prescription indications were not collected, the appropriateness of the use of topical anti-infectives was not evaluated.

According to the recommendations of the clinical practice guidelines, the present study concludes that tobramycin, alone or in combination with dexamethasone, is the most used topical ocular anti-infective in Galicia. It also demonstrates that the use of topical ocular anti-infectives varies by province in our region. This study has also found that the use of this category of medications remained stable during the period 2020–2023, without identifying significant seasonal fluctuations. This study contributes to understanding the use of topical ocular anti-infectives in Galicia and serves as a basis for future research that could include, for example, in-hospital and outpatient prescriptions, stratification by age and sex, indications, dosage and duration of treatment. By developing stewardship measures and understanding the use patterns of these types of medicines, we can prevent the emergence of resistance and lay the foundation for their judicious and sensitive use.

## Data Availability

The raw data supporting the conclusions of this article will be made available by the authors on request.

## Supplementary Materials

The following supporting information can be downloaded at: https://www.mdpi.com/article/10.3390/diseases12100256/s1, Figure S1: Monthly evolution of the number of packages of topical ocular anti-infectives dispensed in Galicia (Spain) between 2020 and 2023. Figure S2: Annual evolution of the number of packages of topical ocular anti-infectives dispensed in Galicia (Spain) between 2020 and 2023. Table S1: Average number of packages of topical ocular anti-infectives per 10,000 inhabitants and year dispensed in Galicia (Spain) between 2020 and 2023.
