# Supplementary material for: An Analysis of the Use of Topical Ocular Anti-Infectives in Galicia (Spain) between 2020 and 2023
Source: Diseases. 2024 Oct 17;12(10):256. doi: 10.3390/diseases12100256 (PMC11507465; doi:10.3390/diseases12100256)
Supplement: Supplementary file 1 [file diseases-12-00256-s001.zip › diseases-3240996-supplementary.pdf]

Figure S1. Monthly evolution of the number of packages of topical ocular anti-infectives dispensed in Galicia (Spain) between 2020 and 2023.

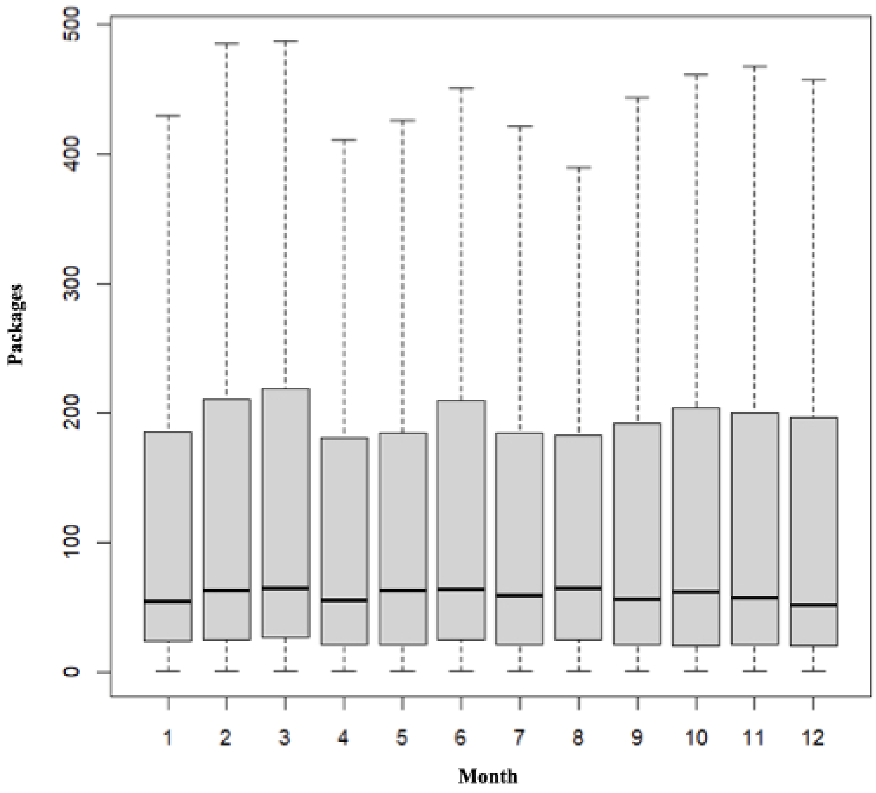

Figure S2. Annual evolution of the number of packages of topical ocular anti-infectives dispensed in Galicia (Spain) between 2020 and 2023.

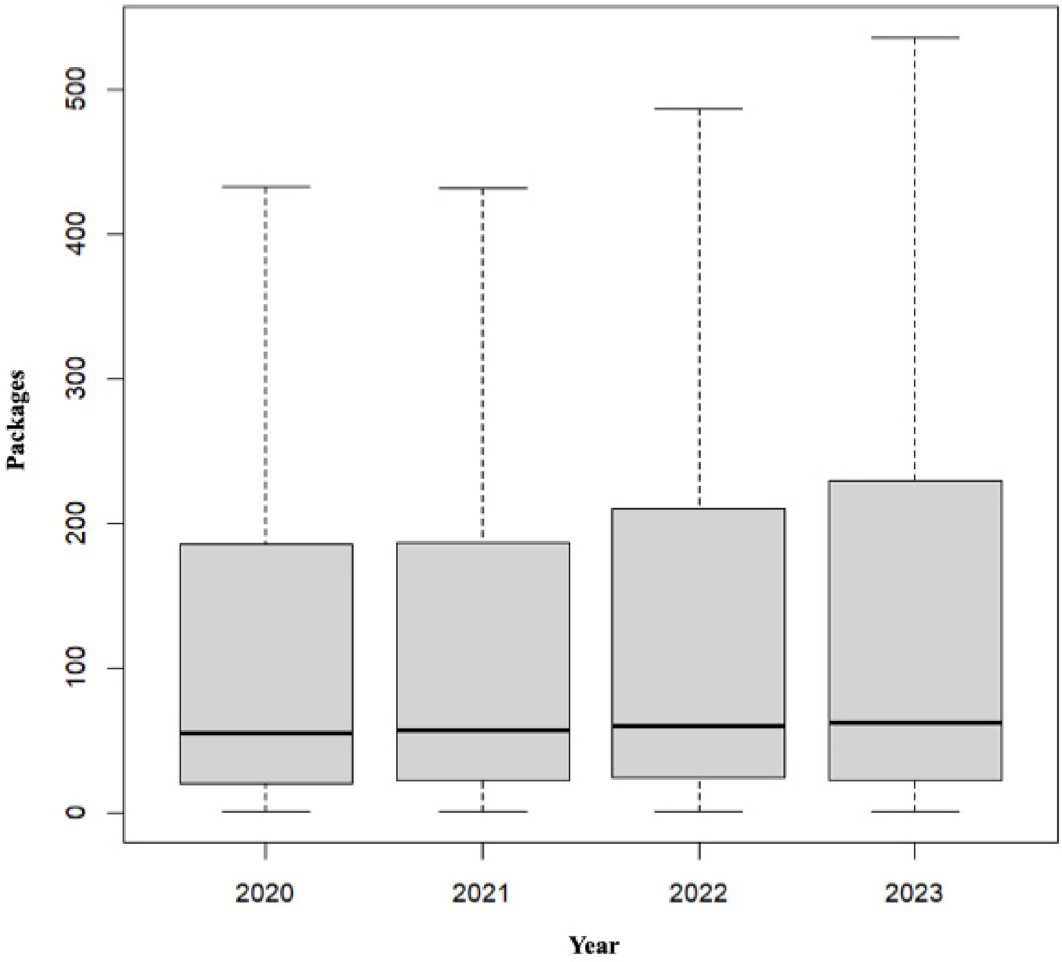

Tabla S1. Average number of packages of topical ocular anti-infectives per 10,000 inhabitants and year dispensed in Galicia (Spain) between 2020 and 2023.

**Virtual Medicinal Product Pack (VMPP)**	**Packages/10,000**
DEXAMETHASONE/TOBRAMYCIN 1 MG/ML + 3 MG/ML EYE DROPS 5 ML	16.95336783
GRAMICIDIN/NEOMYCIN/POLYMIXIN B 25 IU/ML + 1,700 IU/ML + 5,000 IU/ML EYE DROPS 5 ML	8.53407340
TOBRAMYCIN 3 MG/ML EYE DROPS 5 ML	7.63917077
ERYTHROMYCIN 5 MG/G OPHTHALMIC OINTMENT 3.5 G	5.21517340
CIPROFLOXACIN 3 MG/ML EYE DROPS 5 ML	4.09308954
TOBRAMYCIN 3 MG/G OPHTHALMIC OINTMENT 3.5 G	2.62936790
OFLOXACIN 3 MG/ML EYE DROPS 5 ML	2.08409875
MOXIFLOXACIN 5 MG/ML EYE DROPS 5 ML	1.83115473
FUSIDIC ACID 10 MG/G OPHTHALMIC GEL 5 G	1.37960391
GENTAMICIN 3 MG/G OPHTHALMIC OINTMENT 5 G	1.19265592
CIPROFLOXACIN 3 MG/G OPHTHALMIC OINTMENT 3.5 G	1.09677295
CHLORTETRACYCLINE 5 MG/G OPHTHALMIC OINTMENT 3 G	0.89893720
GENTAMICIN 3 MG/ML EYE DROPS 10 ML	0.84839527
OXYTETRACYCLINE 10 MG/G OPHTHALMIC OINTMENT 3.5 G	0.84676733
AZITHROMYCIN 15 MG/G EYE DROPS 0.25 G 6 SINGLE DOSE PACKAGING	0.81993539
ACICLOVIR 30 MG/G OPHTHALMIC OINTMENT 4.5 G	0.60913534
TOBRAMYCIN 3 MG/ML EYE DROPS 4 ML	0.23150186
NETILMICIN 3 MG/ML EYE DROPS 0.3 ML 15 SINGLE DOSE PACKAGING	0.15709147
NETILMICIN 3 MG/ML EYE DROPS 5 ML	0.14534390
CIPROFLOXACIN 3 MG/ML EYE DROPS 0.25 ML 20 SINGLE DOSE PACKAGING	0.13243994
GANCICLOVIR 1.5 MG/G OPHTHALMIC GEL 5 G	0.11691288
DEXAMETHASONE/LEVOFLOXACIN 1 MG/ML + 5 MG/ML EYE DROPS 5 ML	0.08191326
CHLORAMPHENICOL 10 MG/G OPHTHALMIC OINTMENT 3 G	0.07688079
NORFLOXACIN 3 MG/ML EYE DROPS 5 ML	0.05487503
